# Supplementary figures and images for: Optimizing methods for virome analysis based on studies of a synthetic viral community
Source: bioRxiv. 2025 Oct 23:2025.10.23.683462. Preprint. [Version 1] doi: 10.1101/2025.10.23.683462 (PMC12633245; doi:10.1101/2025.10.23.683462)

**Figure S1**

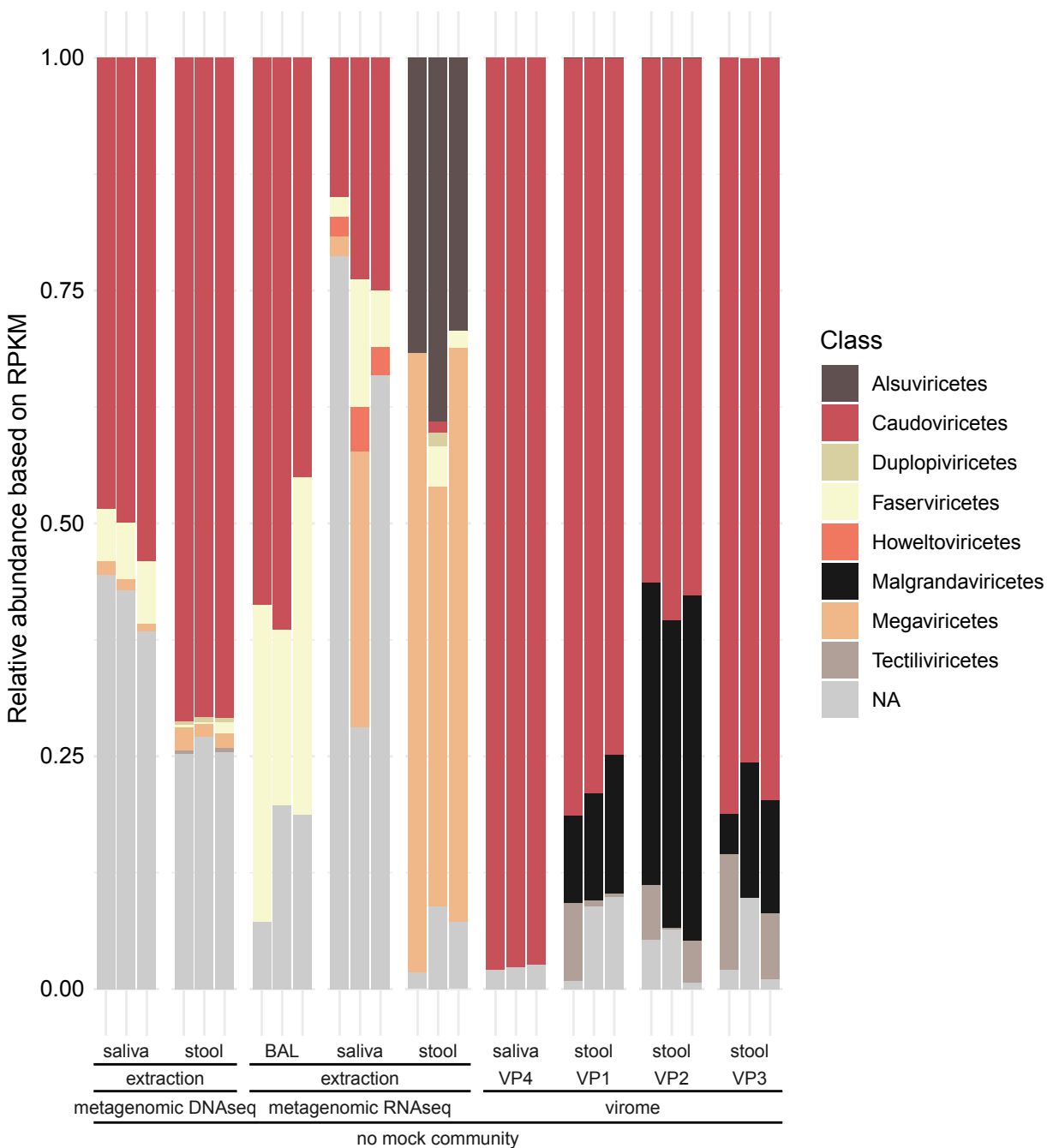

**Figure S2**

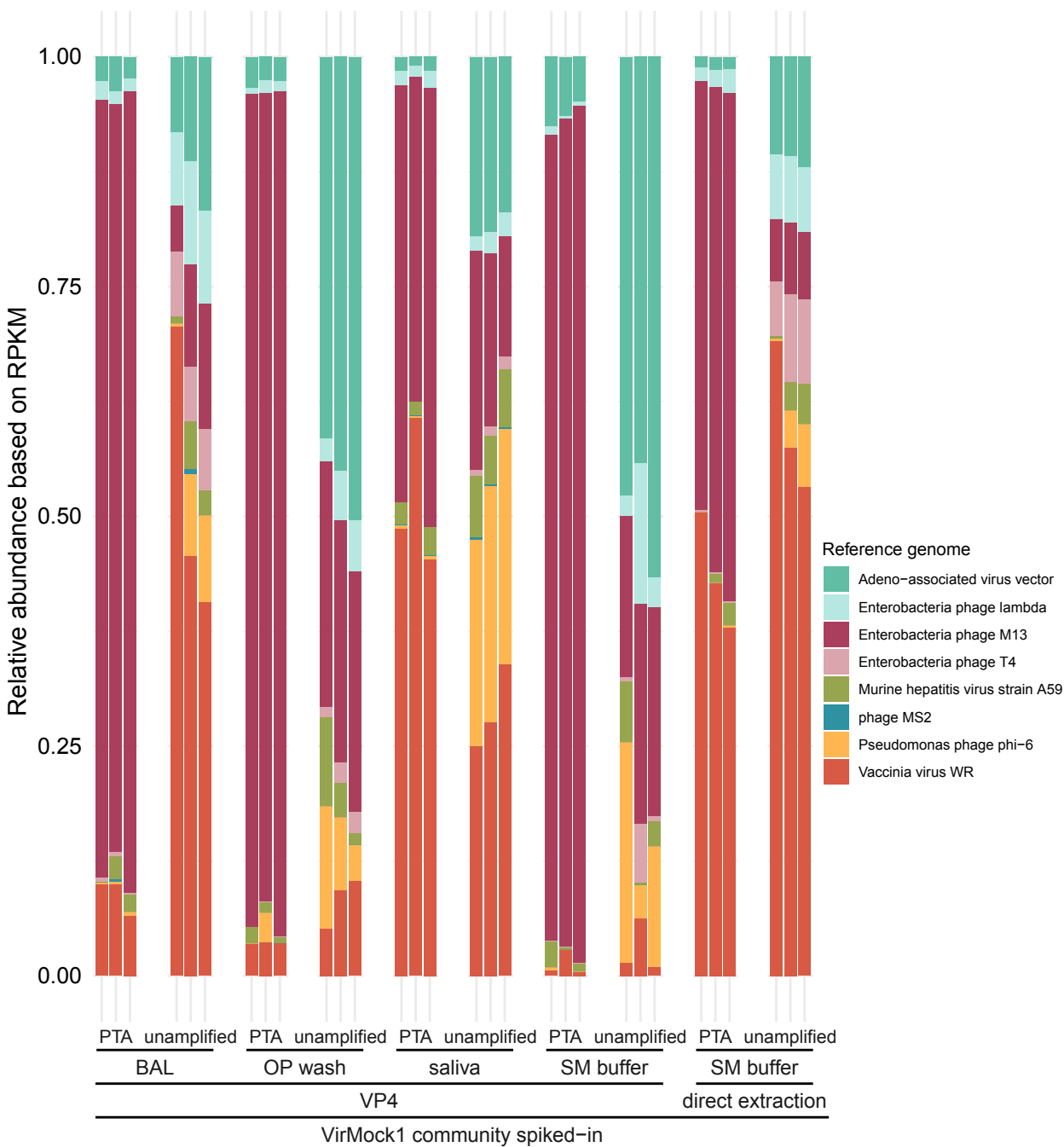

**Figure S3**

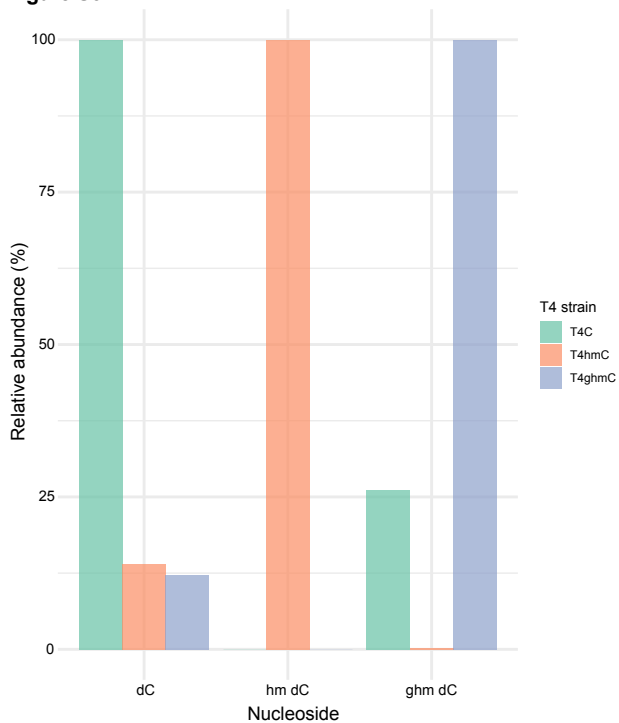

**Figure S4**

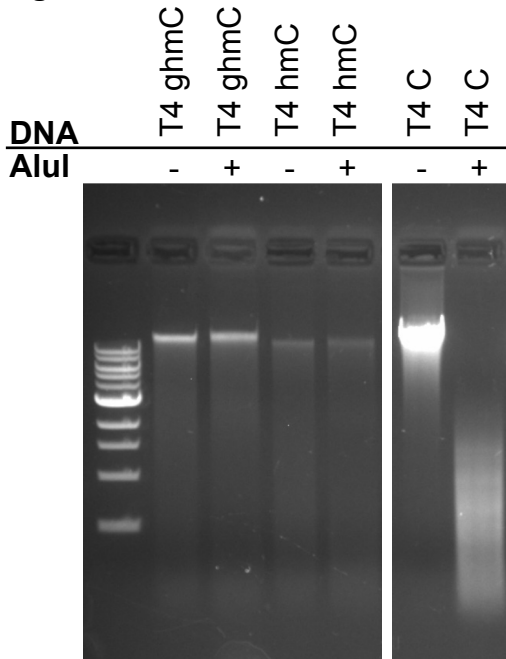

Supplement: Supplement 3 — Figure S1. Relative abundance of class of viruses as annotated by Cenote-Taker2 in saliva, stool or BAL that was analyzed by direct extraction of DNA (red) or RNA (blue), or after viral particle enrichment and analysis using VP1, VP2, VP3, or VP4. Figure S2. Relative abundance of each reference virus after VirMock1 was spiked into saliva, OP wash, or BAL and followed by VP4 and amplification by PTA or remained unamplified. Figure S3. Validation of DNA modifications in T4 strains by LC-MS. Relative abundance of each modified and unmodified nucleoside in T4 C, T4 hmC, and T4 ghmC determined by LC-MS are shown in the bar graph. Figure S4. Validation of T4 strains by AluI digestion of T4 DNA. Phage T4 ghmC, T4 hmC and T4 C DNA left untreated (−) or treated (+) with the restriction enzyme AluI, which cleaves unmodified DNA. [file media-3.pdf]
